# Supplementary material for: Rapid Identification and Analysis of Ochratoxin-A in Food and Agricultural Soil Samples Using a Novel Semi-Automated In-Syringe Based Fast Mycotoxin Extraction (FaMEx) Technique Coupled with UHPLC-MS/MS
Source: Molecules. 2023 Feb 2;28(3):1442. doi: 10.3390/molecules28031442 (PMC9921652; doi:10.3390/molecules28031442)
Supplement: Supplementary file 1 [file molecules-28-01442-s001.zip › molecules-2153133-supplementary.pdf]

Supporting Information

Article

Rapid Identification and Analysis of Ochratoxin-A in Food and Agricultural Soil Samples Using a Novel Semi-Automated In-Syringe Based Fast Mycotoxin Extraction (FaMEx) Technique Coupled with UHPLC-MS/MS

Karthikeyan Prakasham [1], Swapnil Gurrani [1], Jentaie Shiea [1,2,3], Ming-Tsang Wu [1,4], Chia-Fang Wu [1,5], Yi-Jia Ku [6], Tseng-Yu Tsai [6], Hung-Ta Hua [6], Yu-Jia Lin [6], Po-Chin Huang [1,7], Gangadhar Andaluri [8] and Vinoth Kumar Ponnusamy [1,2,3,9,*]

[1]Research Center for Precision Environmental Medicine, Kaohsiung Medical University (KMU), Kaohsiung City-807, Taiwan

[2] Department of Medicinal and Applied Chemistry, Kaohsiung Medical University (KMU), Kaohsiung City-807, Taiwan

[3]Department of Chemistry, National Sun Yat-Sen University, Kaohsiung City-804, Taiwan

[4] Department of Family Medicine, Kaohsiung Medical University Hospital, Kaohsiung Medical University (KMU), Kaohsiung City 807, Taiwan

[5]International Master Program of Translational Medicine, National United University, Miaoli, Taiwan

[6]Research and Development Center, Great Engineering Technology (GETECH) Corporation, No.392, Yucheng Rd., Zuoying District., Kaohsiung City, 813 Taiwan

[7]National Institute of Environmental Health Sciences, National Health Research Institutes (NHRI), Miaoli County 35053, Taiwan

[8]Civil and Environmental Engineering Department, College of Engineering, Temple University, Philadelphia, United States

[9]Department of Medical Research, Kaohsiung Medical University Hospital (KMUH), Kaohsiung City-807, Taiwan

***Corresponding Authors' Email ID: kumar@kmu.edu.tw (Prof. Vinoth Kumar)**

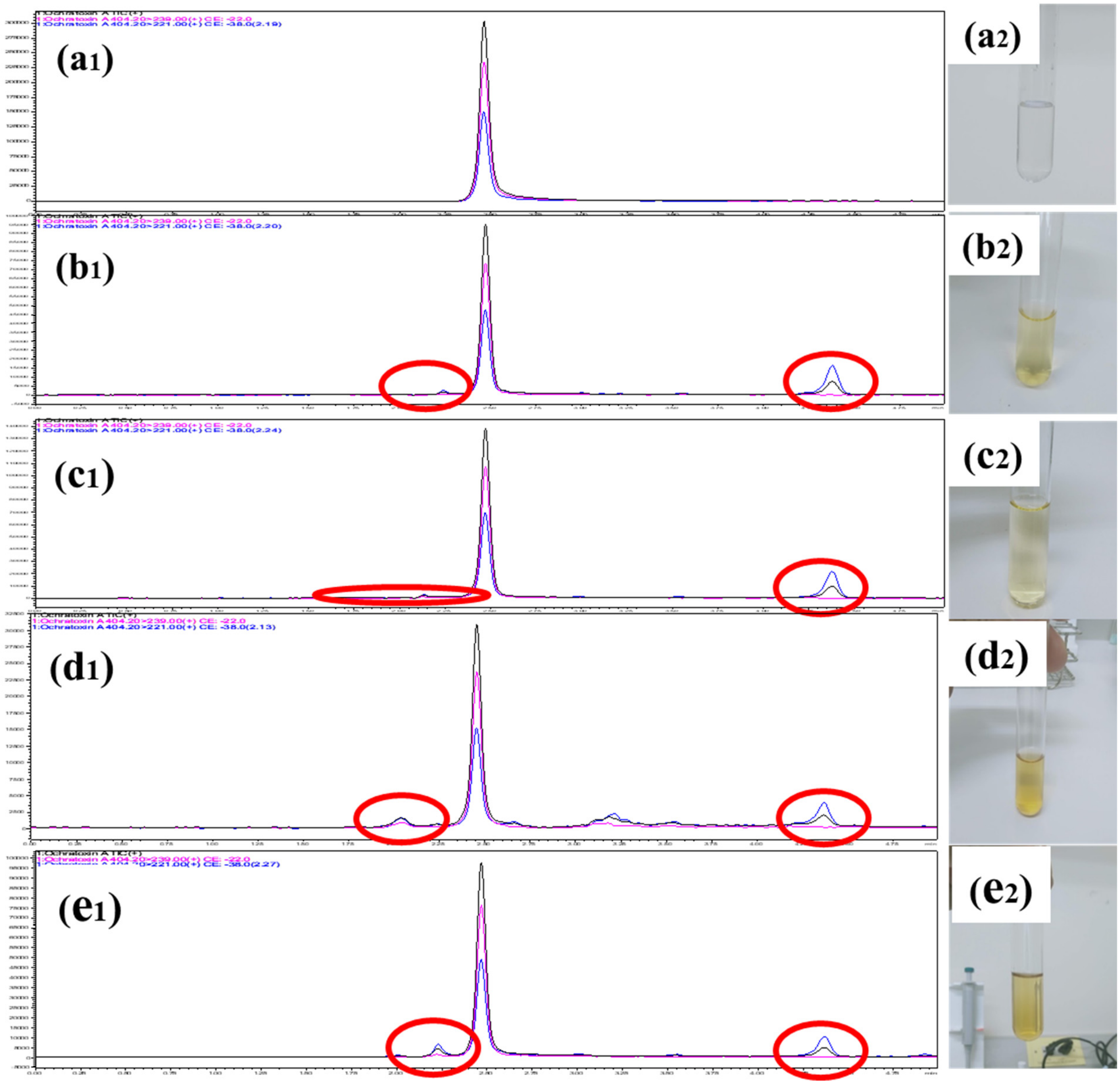

Figure S1, (a1-e1) TIC Chromatogram of OTA extracted from coffee using acetonitrile, acetone, ethyl acetate, methanol, and isopropyl alcohol. (a2-e2) Color of coffee extracts from acetonitrile, acetone, ethyl acetate, methanol, and isopropyl alcohol.

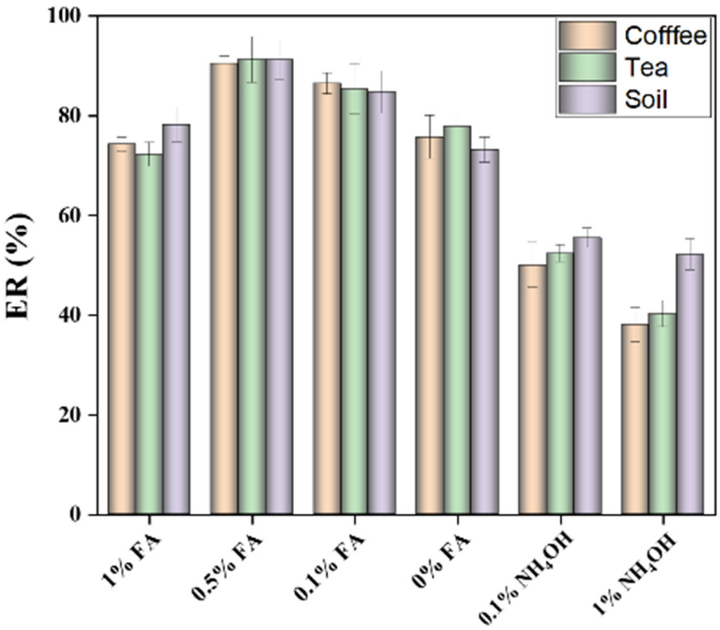

Figure S2: Effect of various pH mediums for the extraction of Ochratoxin A from coffee, tea, and soil samples. Extraction conditions: 250 mg coffee, 2.0 mL solvent (under different pH conditions), 2.5 min of vortex extraction, and clean-up under the flow rate of 0.5ml/min using 1000 mg $MgSO_4$, 75 mg AC and 50 mg C18 with three replicative analysis.
